# The causality between gut microbiota and endometriosis: a bidirectional Mendelian randomization study

**DOI:** 10.3389/fmed.2024.1434582

**Published:** 2024-11-22

**Authors:** Hua Yang

**Affiliations:** Department of Gynecology, The Fifth Affiliated Hospital of Sun Yat-sen University, Zhuhai, China

**Keywords:** endometriosis, gut microbiota, Mendelian randomization, genome-wide association study, causality

## Abstract

**Background:**

Observational studies and animal experiments had suggested a potential relationship between gut microbiota abundance and pathogenesis of endometriosis (EMs), but the relevance of this relationship remains to be clarified.

**Methods:**

We perform a two-sample bidirectional Mendelian randomization (MR) analysis to explore whether there is a causal correlation between the abundance of the gut microbiota and EMs and the direction of causality. Genome-wide association study (GWAS) data ukb-d-N80, finn-b-N14-EM, and MiBinGen were selected. Inverse variance weighted (IVW), weighted median, and MR Egger are selected for causal inference. The Cochran Q test, Egger intercept test, and leave-one-out analysis are performed for sensitivity analyses.

**Results:**

In the primary outcome, we find that a higher abundance of class Negativicutes, genus *Dialister*, genus *Enterorhabdus*, genus *Eubacterium* xylanophilum group, genus *Methanobrevibacter* and order Selenomonadales predict a higher risk of EMs, and a higher abundance of genus *Coprococcus* and genus *Senegalimassilia* predict a lower risk of EMs. During verifiable outcomes, we find that a higher abundance of phylum Cyanobacteria, genus *Ruminococcaceae* UCG002, and genus *Coprococcus* 3 predict a higher risk of EMs, and a higher abundance of genus *Flavonifracto*, genus *Bifidobacterium*, and genus *Rikenellaceae* RC9 predict a lower risk of EMs. In primary reverse MR analysis, we find that EMs predict a lower abundance of the genus *Eubacterium* fissicatena group, genus *Prevotella7*, genus *Butyricicoccus*, family *Lactobacillaceae*, and a higher abundance of genus *Ruminococcaceae* UCG009. In verifiable reverse MR analysis, we find that EMs predict a lower abundance of the genus *Ruminococcaceae* UCG004 and a higher abundance of the genus *Howardella*.

**Conclusion:**

Our study implies a mutual causality between gut microbiota abundance and the pathogenesis of EMs, which may provide a novel direction for EMs diagnosis, prevention, and treatment, may promote future functional or clinical analysis.

## Highlights:

• This study identifies specific GM taxa causally linked to EMs, and conversely, demonstrates that EMs causally influences certain gut microbiota taxa.
• Analysis of GM taxa may contribute to the non-invasive early detection of EMs.
• The GM represents a novel and promising avenue for the screening, treatment, and prevention of endometriosis.

## 1 Introduction

Endometriosis (EMs) is a prevalent condition characterized by the attachment, proliferation, and penetration of viable endometrial tissue outside the uterus, which can lead to chronic pain, reduced fertility, and the formation of nodules or masses due to recurrent bleeding and inflammation. Affecting approximately 10% of women in their reproductive years, the global incidence of endometriosis is estimated at around 196 million (1–3). The treatment for this estrogen-dependent and currently incurable condition typically focuses on alleviating symptoms, as even surgical removal combined with hormonal therapy does not guarantee immunity from recurrence. Moreover, the physical and psychological toll on women before menopause contributes to a significant socioeconomic burden. Surgical intervention with histological verification remains the “gold standard” for diagnosis, as non-invasive methods are yet to be established, despite ongoing investigation into various biomarkers (4–6). The complex etiology and pathogenesis of endometriosis have been subjects of extensive research (7, 8), with the theory of retrograde menstruation being widely accepted but insufficient to explain the entirety of the disease’s biological mechanisms (9). Alternative hypotheses, such as the presence of embryonic Müllerian duct remnants (10), celomic metaplasia (11), and vascular or lymphatic metastasis (12), along with the influence of eutopic endometrium (13), have been proposed to supplement and refine the understanding of EMs. However, a definitive causal link has not been conclusively identified. The prevailing view suggests that EMs is likely caused by an intricate interplay of genetic, epigenetic, hormonal, environmental, and immunological determinants (14).

The GM is defined as the collective microbial inhabitants of the intestine, essential to health and playing pivotal roles in multiple physiological processes, including metabolism, detoxification, nutrient absorption, and the maintenance of homeostasis in the intestinal mucous barrier, immune systems, and endocrine systems (15–18). Perturbations in the composition and abundance of gut microbiota can lead to damage of the mucosal barrier, translocation of bacteria and endotoxins (19), elicitation of various inflammatory responses (20), compromise of the immune milieu (21), and alterations to the metabolome (22). Intestinal dysbiosis not only locally affects the gastrointestinal tract but also elicits systemic responses and has been suggested to correlate with an array of immune or metabolic diseases, such as Graves’ disease (23), multiple sclerosis (24), diabetes (25), systemic lupus erythematosus (26), reproductive disorders (27), and cancers (28–31). Notably, certain bacteria within the gut microbiota carry genes encoding estrogen-metabolizing enzymes, which may regulate circulating estrogen levels (32). Given that estrogen is directly linked to the onset and progression of EMs, it is speculated that the gut microbiota could play a crucial role in EMs.

Although the etiological and risk factors for EMs are largely unknown, recent studies (33–35) have highlighted notable variations at the genus level, with elevated levels of Prevotella, Blautia, and Bifidobacterium, and reduced levels of Paraprevotella, Ruminococcus, and Lachnospira in patients with EMs compared to healthy controls. In the context of patients undergoing abdominal hysterectomy, there has been an observable shift in the microbial composition, particularly a marked increase in the Proteobacteria phylum from 34.36% pre-surgery to 54.04% post-surgery (36). In a mouse EMs model with intraperitoneal injection of endometrial fragments, Ni et al. (37) found that EMs was significantly linked to alternative GM abundance. Chadchan et al. (38) found that metronidazole and broad-spectrum antibiotics could reduce EMs growth in a surgical mouse model. In Rhesus monkeys with EMs, Birney (39) also found significant alterations in the GM between EMs and healthy controls; EMs was related to a higher abundance of gram-negative bacteria and a lower abundance of *Lactobacilli.* A similar correlation had been found in human studies. Shan et al. (40) found that the alpha diversity of GM and the *Firmicutes/Bacteroidetes* ratio were statistically different between stage III/IV EMs and healthy controls. Ata et al. (41) found that compared to healthy women, stage III/IV EMs had an elevated ratio of *Shigella/Escherichia* in their stool. Svensson et al. (42) also found lower alpha diversities, beta diversities, and the ratio of *Firmicutes/Bacteroidetes* in EMs patients. Although these studies suggest that the GM is correlated with EMs, the real effect and impact on EMs are largely unknown. The causal relationship between GM and EMs had been insufficiently addressed owing to the limitations of conventional observational studies that were susceptible to potential confounding bias or reverse causal bias, our research primarily focuses on analyzing the microbial composition at different taxonomic levels, ranging from phylum to species, to understand their role in EMs. By examining these diverse taxonomic ranks, we aim to uncover patterns and correlations that may contribute to our understanding of microbial influence on EMs.

Mendelian randomization (MR) analysis is a sophisticated epidemiological statistical methodology that circumvents the inherent limitations of conventional observational studies. It offers a powerful approach to mitigate the influence of confounding variables and the potential for reverse causation, which often plague such research. This is achieved by leveraging germline single nucleotide polymorphisms (SNPs), which are randomly assigned at conception, to calculate the causal relationship between an exposure and an outcome of interest. The current investigation employs a dual-sample, bidirectional Mendelian randomization analysis to robustly assess the causal nature of these interactions, thereby contributing to our understanding of the complex interplay between the GM and EMs.

## 2 Materials and methods

### 2.1 Inclusion criteria

(1) Human subjects only: Data must be derived from human participants to ensure relevance to the study’s focus on EMs in humans.
(2) Genome-Wide Association Studies (GWAS) databases: Only data from publicly available GWAS databases will be included, specifically focusing on those that compare Single Nucleotide Polymorphisms (SNPs) between individuals with EMs and healthy controls.
(3) Language and time restrictions: There are no language or time restrictions applied to the selection of studies, allowing for a comprehensive review of available literature.
(4) Population-scale cohorts: Studies should include population-scale cohorts with sufficient sample sizes to ensure statistical power in detecting associations between SNPs and endometriosis risk.
(5) High-density SNP arrays: Studies must have utilized high-density genome-wide SNP arrays for genotyping to ensure the accuracy and comprehensiveness of the genetic data.
(6) European descent: This study focuses on individuals of European descent to maintain consistency in the genetic background across the samples analyzed.
(7) Healthy controls: Studies must include non-gender-specific health controls without any diagnosed endometriosis to serve as a comparison group for identifying genetic differences associated with the disease.

### 2.2 Exclusion criteria

(1) Preclinical or animal models: Data obtained from preclinical studies or animal models will be excluded, as the focus is on human genetic associations with endometriosis.
(2) Non-GWAS data: Studies that do not employ a GWAS approach or do not compare SNPs between cases and controls will be excluded.
(3) Insufficient sample size: Studies with inadequate sample sizes, which may limit the ability to detect significant associations, will be excluded.
(4) Lack of control group: Studies lacking a proper control group of healthy individuals without endometriosis will not be considered.

### 2.3 Genome-wide association study (GWAS) statistics of EMs

The GWAS databases included for the study compared SNPs between individuals with EMs and healthy controls without language or time restrictions, excluding data from preclinical or animal models. After evaluation, two major public mete-datasets on EMs were selected: ukb-d-N80 (43): includes 9,983,671 SNPs, with 1,496 EMs cases and 359,698 non-gender-specific health controls of European descent, and finn-b-N14-EMs (44): comprises 16,377,306 SNPs, with 8,288 EMs cases and 68,969 non-gender-specific health controls also of European descent.

### 2.4 GWAS statistics of gut microbiota

The GWAS data on GM, MiBioGen (45), was published in 2021, which has amassed 18 population-scale cohorts comprising approximately 19,000 individuals. This initiative seeks to generate novel insights for the burgeoning field of microbiome research. Each participating cohort has conducted comprehensive surveys of the gut microbiota utilizing 16S rRNA gene sequencing and has performed genotyping on their participants using high-density genome-wide SNP arrays. In total, 197 taxa were included (comprising 9 phyla, 16 classes, 19 orders, 33 families, and 120 genera), and 14 unknown taxa (11 genera and 3 families) were excluded.

### 2.5 Instrumental variable selection

GM is analyzed in distinct independent taxa. To ensure the robustness and veracity of the analysis results, several optimization strategies are used to extract closely related instrumental variables (IVs) (28, 46–48). Initially, a strong statistical threshold of *p* < 5 × 10^–8^ is set to extract SNPs intensively correlated with the GM. However, since no SNPs meet this criterion for most taxa, a second threshold of *p* < 5 × 10^–6^ is adopted for MR analysis. Minor allele frequency (MAF) threshold = 0.01 is set to filter common SNP mutations. To avoid bias caused by LD among IVs, an R-squared (R^2^) value less than 0.001 and a clumping distance of 10,000 kilobases (kb) are used as thresholds to clump SNPs with LD. The horizontal pleiotropy of the SNPs is tested using MR-PRESSO. Outlier tests compute *p*-values for individual significant pleiotropy, while global tests compute *p*-values for overall significant pleiotropy. SNPs are ranked by increasing *p*-values and removed sequentially. The MR-PRESSO global test recalculates the *p*-value for the remaining SNPs until it exceeds 0.05. We also calculate F statistics to avoid weak IVs bias. The formula used was F = R^2^ × (N-1-K)/(1-R^2^) × K, R^2^ represents the coefficient of determination, which indicates the proportion of the variance in the dependent variable that is predictable from the independent variables. N is the sample size, or the total number of observations. K is the number of independent variables in the regression model. The term (N-1-K) represents the degrees of freedom for the regression model. The term (1-R^2^) represents the proportion of variance that is not explained by the regression model. The term K in the denominator represents the degrees of freedom for the residuals. The F-statistic is calculated by multiplying R^2^ by the ratio of the regression degrees of freedom to the residual degrees of freedom, adjusted for the unexplained variance. This value is used to test the null hypothesis that all coefficients in the population regression model are equal to zero. A higher F-statistic value suggests that the model explains more of the variance in the dependent variable and is less likely to be due to random chance. Where SNPs with *F*-values below 10 were discarded in subsequent MR analyses. We check the genotype-pheotype associations during website PhenoScanner for each SNP, those SNPs related with potential confounding factor of EMs are removed. The remaining SNPs are then used for subsequent MR analysis. These strategies aim to ensure that the SNPs effectively influence both the exposure (GM) and the outcome (EMs), maintaining the validity of the MR analysis.

### 2.6 Mendelian randomization analysis

The causal correlation between GM and EMs is inferred using a bidirectional two-sample Mendelian randomization (MR) analysis. The following steps are undertaken:

Selection of SNPs for GM: SNPs closely associated with gut microbiota are selected from the GWAS data to test for a causal effect on EMs.

Selection of SNPs for EMs: SNPs closely associated with EMs are used as exposure variables in the reverse MR analysis, with the abundance of gut microbiota as the outcome to test if EMs have an effect on altering the gut microbiota.

MR methods: Three main MR methods are employed for the analysis of multiple SNPs: Inverse-variance weighted (IVW) method, this method is considered more robust than the other methods and thus the primary reliance for MR results. Weighted median estimator (WME). MR-Egger regression. The Wald ratio test (49) is applied when only one SNP is included in the analysis to evaluate the association between gut microbiota taxa and EMs.

Sensitivity tests: These are conducted to assess the reliability of the findings: Leave-one-out test (50): Used to determine if the causal correlation is due to a single SNP. Causal direction test: Compares the variance caused by the SNPs in the exposure to that in the outcome to establish directional robustness. F-statistics (51): Calculated to identify weak instrumental variables (IVs), where an *F*-value less than 10 indicates a weak IV and leads to its exclusion from subsequent MR analysis.

Software: All analyses are performed using R for Windows version 4.3.0, utilizing the “TwoSampleMR” package for the MR analysis and the “MR-PRESSO” package for testing horizontal pleiotropy.

### 2.7 Heterogeneity

Cochran’s Q statistic (52) is utilized to test for heterogeneity among the instrumental variables. A *Q*-value greater than the number of SNPs minus one or a *p*-value less than 0.05 suggests heterogeneity and invalid IVs.

**FIGURE 1:**
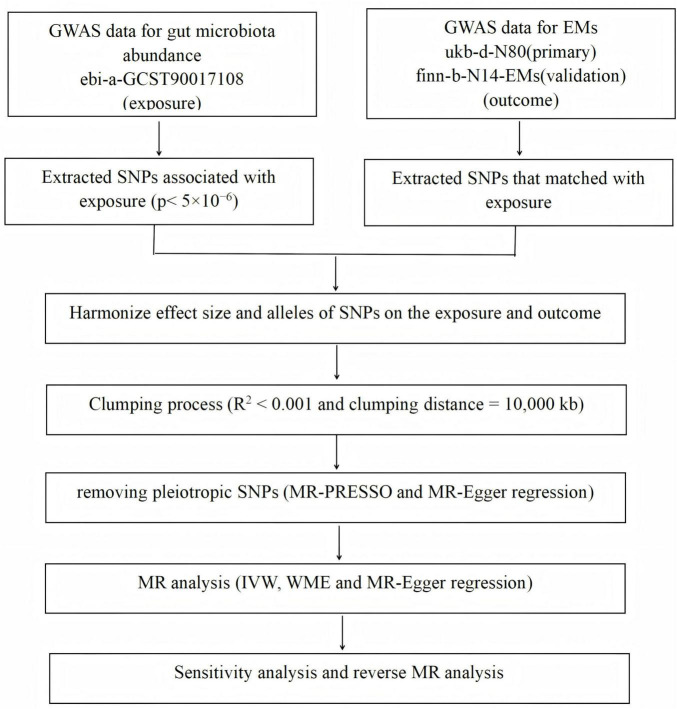
The flowchart of the present mendelian randomization (MR) analysis.

The flowchart detailing the MR analysis process is presented in Figure 1.

## 3 Results

### 3.1 SNP selection

In the first step of the analysis, SNPs associated with individual GM taxa are extracted. A total of 1 to 11 SNPs are associated with each of the 197 taxa (comprising 9 phyla, 16 classes, 19 orders, 33 families, and 120 genera) at a significance level of *p* < 5 × 10^–6^. This selection is based on the optimization strategies previously outlined. The number of SNPs per taxon is detailed in Supplementary Table 1. No pleiotropic effects are identified by the MR-PRESSO global test (*p* > 0.05).

### 3.2 Primary causal correlation of GM on the risk of EMs

**FIGURE 2:**
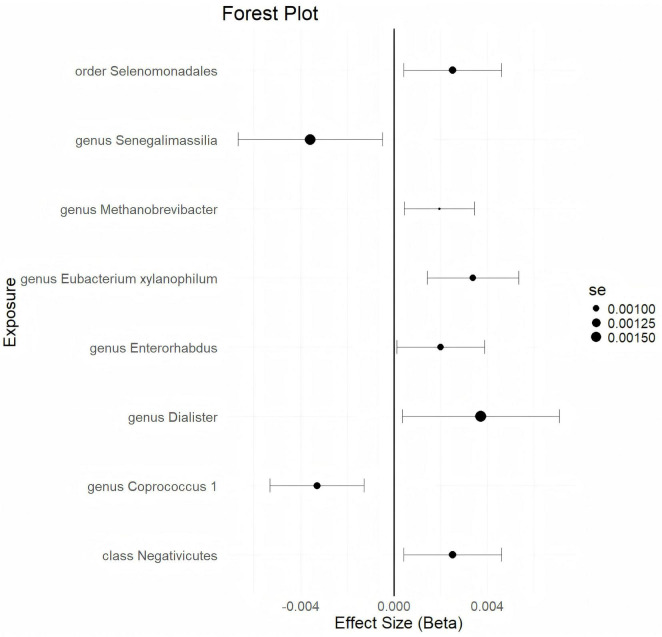
The forestplot summarized the causality of gut microbiota on the risk of endometriosis during Genome wide association study (GWAS) data: ukb-d-N80.

Using a statistical threshold of *p* < 5 × 10^–6^ and with GWAS data from ukb-d-N80 as the outcome, the analysis reveals that a higher abundance of the *class Negativicutes* is causally linked to a higher risk of EMs (*b* = 0.002521, *p* = 0.01863 by IVW test) (Figure 2). Homogeneous results are obtained by MR Egger and Weighted median tests, with no horizontal pleiotropy (*p* = 0.359) or heterogeneity (*p* = 0.4014) detected among the SNPs. The causal direction analysis shows that the variance explained in exposure is significantly stronger than in the outcome (*p* = 1e-36), and the leave-one-out test confirms that the causality is not driven by a single SNP. These findings suggest that the causal relationship between the *class Negativicutes* and EMs is robust. Additionally, higher abundances of the *genus Dialister, genus Enterorhabdus, genus Eubacteriumxylanophilum, genus Methanobrevibacter*, and *order Selenomonadales* are found to causally predict a higher risk of EMs (Figure 2 and Supplementary Table 2). Conversely, a higher abundance of the *genus Coprococcus 1* causally predicts a lower risk of EMs (*b* = -0.003294, *p* = 0.001354 by IVW test) (Figure 2), with homogeneous results from MR Egger and Weighted median tests, no horizontal pleiotropy (*p* = 0.657), no heterogeneity (*p* = 0.5713), and a strong significant variance explained in exposure over outcome (*p* = 4.34e-36). The leave-one-out test supports that the causality is not influenced by a single SNP. These results indicate that the causal correlation between the *genus Coprococcus 1* and EMs is robust. Furthermore, a higher abundance of the *genus Senegalimassilia* causally predicts a lower risk of EMs (*b* = -0.003588, *p* = 0.02319 by IVW test) (Figure 2). However, there are not enough SNPs (*n* = 2) to perform the MR Egger and Weighted median tests.

### 3.3 Verified causal correlation of GM on the risk of EMs

**FIGURE 3:**
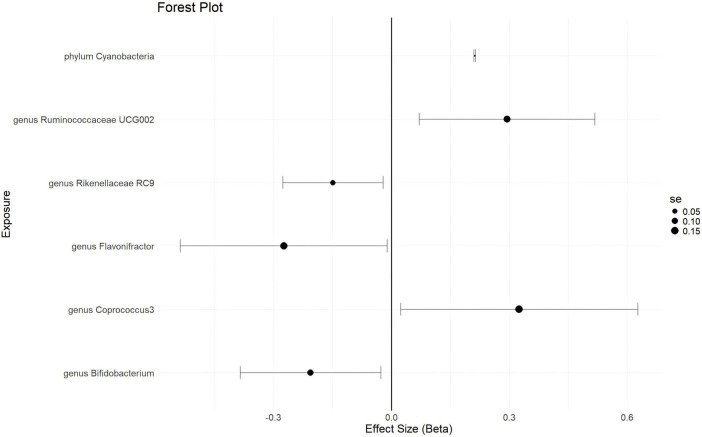
The forestplot summarized the causality of gut microbiota on the risk of endometriosis during Genome wide association study (GWAS) data: finn-b-N14-EMs.

With a statistical threshold set at *p* < 5 × 10^–6^ and using GWAS data from finn-b-N14-EMs as the outcome, the analysis shows that a higher abundance of the *phylum Cyanobacteria* is causally linked to a higher risk of EMs (*b* = 0.2114, *p* = 0.03997 by IVW test) (Figure 3). Consistent results are obtained from MR Egger and Weighted median tests, with no horizontal pleiotropy (*p* = 0.359) or heterogeneity (*p* = 0.4014) detected among the SNPs. Although there is not enough data for causal direction analysis, the leave-one-out test indicates that the causality is not influenced by a single SNP. These findings suggest that the causal relationship between the *phylum Cyanobacteria* and EMs is robust. Additionally, a higher abundance of the *genus Ruminococcaceae UCG002* and *genus Coprococcus 3* is found to causally predict a higher risk of EMs (Figure 3 and Supplementary Table 3). Conversely, a higher abundance of the *genus Bifidobacterium* is causally linked to a lower risk of EMs (*b* = -0.2059, *p* = 0.02419 by IVW test) (Figure 3), with consistent results from MR Egger and Weighted median tests, no horizontal pleiotropy (*p* = 0.73), no heterogeneity (*p* = 0.6216), and the leave-one-out test confirming that the causality is not driven by a single SNP. These results suggest that the causal relationship between the *genus Bifidobacterium* and EMs is robust. Furthermore, a higher abundance of the *genus Flavonifractor* and *genus Rikenellaceae RC 9* is found to causally predict a lower risk of EMs (Figure 3 and Supplementary Table 3).

### 3.4 Primary causal correlation of EMs on GM

**FIGURE 4:**
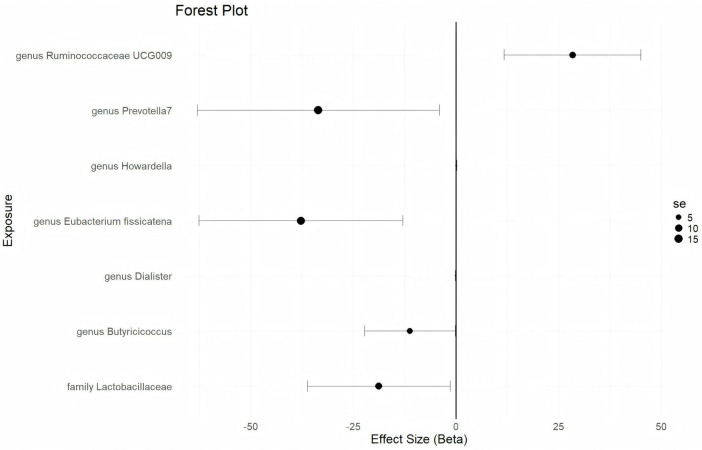
The forestplot summarized the causality of endometriosis on gut microbiota.

In this analysis, with a statistical threshold set at *p* < 5 × 10^–6^, 23 closely related SNPs are extracted as instrumental variables (IVs) for the GWAS data from ukb-d-N80, using GM taxa as the outcome. The results indicate that EMs causally predict a higher abundance of the *genus Ruminococcaceae UCG009* (*b* = 28.39, *p* = 0.0008221 by IVW test) (Figure 4). Consistent findings are observed through MR Egger and Weighted median tests, with no horizontal pleiotropy (*p* = 0.63) or heterogeneity (*p* = 0.635) detected among the SNPs. The causal direction analysis reveals that the variance explained in exposure is not significantly different from the variance explained in the outcome (*p* = 0.285), and the leave-one-out test confirms that the causality is not driven by a single SNP. These findings suggest that the causal relationship between EMs and the increased abundance of the *genus Ruminococcaceae UCG009* is robust. Additionally, EMs are found to causally predict a lower abundance of the *genus Eubacterium fissicatena* (*b* = -28.39, *p* = 0.0008221 by IVW test) (Figure 4), with homogenous results from MR Egger and Weighted median tests, no horizontal pleiotropy (*p* = 0.362), and no heterogeneity (*p* = 0.4528) detected. The causal direction analysis shows that the variance explained in exposure is not significantly different from the variance explained in the outcome (*p* = 0.287), and the leave-one-out test indicates that the causality is not influenced by a single SNP. These results suggest that the causal association between EMs and the reduced abundance of the *genus Eubacterium fissicatena* is robust. Furthermore, EMs are found to causally predict lower abundances of the *genus Prevotella7, genus Butyricicoccus*, and *family Lactobacillaceae* (Figure 4 and Supplementary Table 4).

### 3.5 Verified causal correlation of EMs on GM

With a statistical threshold set at *p* < 5 × 10^–6^, 30 closely related SNPs are used as instrumental variables (IVs) for the GWAS data from finn-b-N14-EMs, using GM taxa as the outcome. The analysis reveals that EMs causally predict a higher abundance of the *genus Howardella* (*b* = 0.1271, *p* = 0.01087 by IVW test) (Figure 4). This finding is supported by consistent results from the MR Egger and Weighted median tests. No horizontal pleiotropy (*p* = 0.275) or heterogeneity (*p* = 0.5403) is found among the SNPs. Although there is not enough data for causal direction analysis, the leave-one-out test indicates that the causality is not influenced by a single SNP. These results suggest that the causal correlation between EMs and an increased abundance of the *genus Howardella* is robust, as illustrated in Figure 4 and Supplementary Table 5. Additionally, EMs are found to causally predict a lower abundance of the *genus Ruminococcaceae UCG004* (*b* = -0.07478, *p* = 0.01742 by IVW test) (Figure 4), with homogeneous results from the MR Egger and Weighted median tests. No horizontal pleiotropy (*p* = 0.391) or heterogeneity (*p* = 0.4917) is detected among the SNPs. While there is insufficient data for causal direction analysis, the leave-one-out test confirms that the causality is not affected by a single SNP. These results suggest that the causal association between EMs and a decreased abundance of the *genus Ruminococcaceae UCG004* is robust, as shown in Figure 4 and Supplementary Table 5.

## 4 Discussion

The present study is the first to employ a bidirectional MR approach to investigate the reciprocal causal relationships between the GM and EMs. This research holds significant potential for guiding clinical practice in the field of microbiome studies. From the largest GWAS datasets on GM and two independent EMs, robustly associated SNPs have been extracted. A thorough genetic correlation analysis of over 400,000 European individuals has led to the discovery that SNPs predisposing to certain GM taxa have a causal relationship with EMs. Conversely, it has also been found that SNPs predisposing to EMs have a causal relationship with specific GM taxa. These findings suggest a new direction for the non-invasive early diagnosis of EMs. Targeting the GM may represent a novel strategy for the prevention, treatment, and long-term management of EMs.

The GM plays a pivotal role in human health, influencing multiple aspects of physiology and immunity. Eubiosis refers to a balanced GM that contributes to host health, whereas dysbiosis indicates an imbalance associated with disease states like EMs. Dysbiosis may promote EMs by increasing intestinal permeability and systemic inflammation, potentially altering immune responses and fostering a pro-inflammatory milieu that facilitates EMs development (53–55). EMs is a very common disease during the childbearing period for females, causing serious health and mental distress. Many of these women experience chronic pelvic pain, infertility, excessive bleeding, and so on. The diagnosis is delayed usually because it can only be definitely diagnosed by invasive methods (56), and curative treatments are unavailable because it is estrogen-dependent. In the past few years, owing to the rapid development of science technologies, omics research, bioinformatics, and high-throughput sequencing technology, a growing body of research had found the potential relationship between gut microbiota and EMs (57–59). In recent decades, despite alterations in gut microbiota had been reported in animal models and females with EMs, the results were inconsistent, and whether there was a causal correlation and the direction of causality between EMs and gut microbiota abundance was unclear.

In this MR study, dual verification is adopted to verify the robustness of causality. For primary analysis, we set GWAS data: ukb-d-N80 (included 1496 cases and 359698 controls from European ancestors) as the outcome, MR results find genetic liability to *class Negativicutes, genus Dialister, genus Enterorhabdus, genus Eubacteriumxylanophilum, genus Methanobrevibacter, order Selenomonadales, genus Coprococcus 1* and *genus Senegalimassilia* causally associate with EMs. For verifiable analysis, we set summary GWAS data: finn-b-N14-EMs(included 8288 cases and 68969 controls from European ancestors) as the outcome, MR results find genetic liability to *phylum Cyanobacteria, genus Ruminococcaceae UCG002, genus Coprococcus3, genus Bifidobacterium, genus Flavonifractor* and *genus Rikenellaceae RC9* causally associate with EMs. Our results suggest that certain GM taxa may be involved in pathogenesis of EMs, and GM analysis may help to identify females at high risk for EMs and may be helpful to diagnose EMs at an earlier time.

EMs pathogenesis contains complex metabolic, genetic, immunological, and immunological alterations. Most recent evidence shows that intercellular crosstalk through micro-RNA has a critical role in EMs. To date, the exact mechanism by which the GM affects EMs is largely unknown. Baker et al. (60) found a vicious cycle between GM and EMs through chronic stress and β-adrenergic signaling, regarded as the “estrogen-gut-brain axis.” Chadchan et al. (38) found that short-chain fatty acids in the gut might affect the gut immune barrier, might regulate the pathogenesis of EMs. Jiang et al. (61) found GM might affect the formation and function of lymphoid structures and immune cells during the intestinal wall, might affect the development of EMs. Due to immunological dysfunction of immunological (62) and estrogen homeostasis (63) playing a key role in the development and progression of EMs, and the potential influence of GM on immune and estrogen levels, researchers speculate that immunological and estrogen mechanisms maybe the key mediators.

The histopathological features of EMs are characterized by local inflammation. An imbalance of the inflammatory reaction and immune system is a crucial cause of EMs. Recent studies had shown a strong relationship between alterations in gut microbiota and psoriasis (64), inflammatory bowel disease (65), arthritis (47), neuropsychiatric diseases (66), and some cancers. These can be partially explained by the immunoregulation of the GM for systemic inflammatory reactions. As unbalanced immune and inflammatory responses are thought to be involved in EMs, the causality between GM and EMs is logically rational. A mouse model found that fecal transplant from EMs mice could alter EMs progression accompanied by modulation of inflammatory and immune responses. Lui et al. (67) found that alteration of GM might influence the composition and function of mucosal T cells (Th1, Treg, Th17, etc.), which might cause an imbalance in the mucosal immune system, further triggering inflammation and disease. Kogut et al. (68) found that alteration of GM could cause elevated levels of systematic immune mediators. Macrophages are the predominant immune cell population in the ascites of EMs and may play an important role in EMs. Elkabets et al. (69), Lobo et al. (70), and Rao et al. (71) found dysfunctional NK cells could damage the phagocytic activity of macrophages and induce Treg lymphocytes, which might promote ectopic endometrial cells to escape from immune surveillance. Recent studies had suggested that alterations in GM abundance might cause inappropriate macrophage activity (72, 73), which might be involved in the pathogenesis of EMs. Unfortunately, due to the limitation of GWAS data on immune cells and immune mediators, we can not explore whether there is a causality between GM and immune systems, or whether the causality between GM abundance and EMs is mediated by immune systems, which is also a crucial implication for further research.

Another potentially critical mediator between GM and EMs is estrogen. Previous research had shown that alterations in the GM might lead to increased circulatory estrogen levels (74, 75). Certain taxa of GM can produce β-glucuronidase or β- βglucosidases involved in estrogen metabolism, which is defined as “estrobolome” (76). Estrogen metabolism mainly occurs in the liver. The liver can inactivate estrogen through sex hormone-binding globulin. The β-glucuronidase or β-glucosidases came from the GM can catalyze the decomposition of conjugated estrogen; thus, estrogen reabsorption from the intestine is upregulated. High-throughput sequencing of gut microbial genome finds multiple bacterial taxa carries the gene coded for β-glucuronidase or β-glucosidases, including *Bacteroid, Bifidobacterium, Escherichia, Lactobacillus*, and *Lactobacillus* (77–79). Yan et al. (80) found that the abundance of *Escherichia* was higher in the stool of patients with EMs than in healthy controls. Yuan et al. (81) also reported a higher abundance of *Bifidobacterium* and *Escherichia* in EMs mouse models. In our MR study, we find genetic liability to the *genus Bifidobacterium* (belonging to the astrobleme) causally associates with EMs, confirming that the GM maybe involved in the pathogenesis of EMs through estrogen metabolism.

Although numerous clinical studies had reported that GM of EMs differed from that of healthy females, the results were inconsistent. Animal studies had found a bidirectional correlation between GM and EM risk (82). Whether GM changed before or after the onset of EMs in the same female has not been clarified yet. Whether EMs can cause alterations in GM is known still, which seems to be difficult to solve by epidemiological or observational studies. Therefore, we adopt a reverse MR study to clarify this puzzle.

During the reverse MR study, we set GWAS data: ukb-d-N80 as exposure first, MR results find SNPs predisposition to EMs causally related to *genus Ruminococcaceae UCG009*, g*enus Eubacterium fissicatena*, *genus Prevotella7*, *genus Butyricicoccus*, and *family Lactobacillaceae*. For verifiable analysis, we set summary GWAS data: finn-b-N14-EMs as exposure, MR results find SNPs predisposition to EMs causally related to *genus Howardella* and *genus Ruminococcaceae UCG004*. Our results suggest that EMs may affect certain GM taxa, indicating that GM analysis maybe a helpful tool for the non-invasive diagnosis of EMs. However, the mechanism by which EMs affect GM is largely unknown, which is a crucial implication for further research.

Prospective studies investigating the relationship between GM and EMs, though challenging, are feasible with rigorous design. Key elements include selecting a diverse cohort of women with EMs and a control group, using strict inclusion and exclusion criteria to minimize confounding factors. Standardizing sample collection and analysis, potentially with advanced sequencing, is crucial for reliability. Integrating immunological assays can elucidate the interplay between microbial shifts and inflammatory processes, potentially revealing causal pathways in EMs development.

Although there are several Mendelian randomization studies (83–86) to explore causal correlation between GM and EMs. Due to the GWAS data came from different population and the lower significance threshold (*P* < 1.0 × 10^–5^), the conclusions are inconsistent. Our study has several strengths:

(1) First bidirectional MR study: Our study is the first to conduct a bidirectional MR analysis exploring the mutual causal correlation between GM and EMs. This novel approach allows for a comprehensive understanding of the reciprocal relationships between these factors.
(2) Largest sample sizes: To date, our research encompasses the largest sample sizes in this field, enhancing the statistical power and generalizability of our findings.
(3) Dual verification: We have employed dual verification methods to ensure the robustness of our results, thereby increasing confidence in the validity of our conclusions.
(4) Elimination of confounding bias: The MR analysis methodology effectively eliminates confounding biases inherent in observational studies, aligning our evidence with that of randomized controlled trials (RCTs).
(5) Strongly associated SNPs: Our study focuses on SNPs that are strongly associated with GM, providing a solid genetic basis for exploring their relationship with EMs.
(6) Comparison with dependent databases: By comparing our findings with two dependent EMs databases, we enhance the reliability and relevance of our results.
(7) No pleiotropy or heterogeneity: Sensitivity analyses indicate no pleiotropy or heterogeneity, reinforcing the statistical robustness of our outcomes.
(8) Potential novel biomarker: Our findings suggest certain GM signatures may act as novel biomarkers for EMs, offering potential for non-invasive diagnostic methods.
(9) Consistency with existing literature: Our findings resonate with existing literature, particularly the review by Iavarone et al. (87), which highlights correlations between GM composition and EMs. Our study further supports the notion that specific microbial signatures could be indicative of pathophysiological states.
(10) Therapeutic implications: Given the accessibility of treatments for GM dysbiosis through prebiotics or probiotics, our results pave the way for novel therapeutic strategies beyond traditional medicines and surgery for EMs management.

Despite the significant contributions of our research, several limitations must be acknowledged.

(1) The sequencing methodology employed relied on 16S rRNA gene analysis, which, while informative, does not provide species-level resolution of GM. This constraint potentially obscures critical details within the endometrial microbial communities that could be pertinent to the pathogenesis of EMs. Achieving species-level resolution through advanced techniques such as shotgun metagenomics or targeted PCR assays could significantly enhance our comprehension by identifying specific microbial taxa associated with EMs and elucidating the underlying pathogenic mechanisms. Future studies should therefore adopt these high-resolution sequencing technologies to delve deeper into the GM composition.
(2) The population utilized in our study is of European descent, raising concerns about the generalizability of our findings to other ethnicities and geographical regions. Ethnic and geographical variations are known to influence GM composition, potentially limiting the applicability of our results to more diverse populations. To address this, future research should include participants from multiple races and geographic locations to ensure broader relevance and validity.
(3) The use of summary data in our GWAS analysis means that individual characteristics were not available for consideration, making it challenging to assess the impact of personalized confounding factors. The absence of individual-level data limits our ability to control for potential confounders that could affect the association between GM and EMs.
(4) Our stringent inclusion criteria may have excluded genetic variants associated with GM that could contribute to EMs risk, potentially leading to missed opportunities for discovery. The rigorous thresholds applied at the IV selection stage might have inadvertently filtered out relevant genetic markers.
(5) Although we analyzed over 200 taxa of GM, only a few showed statistical correlation with EMs. The possibility that these results occurred by chance cannot be entirely dismissed. Therefore, future investigations should aim to enroll larger sample sizes across diverse racial and geographical backgrounds to strengthen causal inferences. There is an urgent need for further in-depth mechanistic studies to understand the precise roles of GM alterations in the development of EMs. Additionally, exploring the diagnostic and therapeutic potential of targeting GM abundance in EMs requires comprehensive evaluation in subsequent research endeavors.

In this study, we have conducted a comprehensive assessment of the relationship between GM and EMs. Our findings indicate that there exists a causal correlation between specific GM taxa and EMs. We identified 14 GM taxa that are causally related to EMs, and conversely, EMs appear to be causally related to seven GM taxa. The bidirectional nature of these findings suggests a mutual causality between the GM and the pathogenesis of EMs. These results offer novel insights into the potential for GM as a diagnostic tool, as well as a target for the prevention and treatment of EMs. The implications of our study could pave the way for future functional and clinical analyses, potentially leading to the development of new therapeutic strategies that leverage the GM to combat EMs. These discoveries may also contribute to a deeper understanding of the complex interplay between the GM and EMs, providing a foundation for further research in this area.

## Data Availability

The original contributions presented in the study are included in the article/Supplementary material, further inquiries can be directed to the corresponding author.
