# Supplementary material for: The causality between gut microbiota and endometriosis: a bidirectional Mendelian randomization study
Source: Front Med (Lausanne). 2024 Nov 22;11:1434582. doi: 10.3389/fmed.2024.1434582 (PMC11621931; doi:10.3389/fmed.2024.1434582)
Supplement: Supplementary file 1 [file Table_1.doc]

Table S1. The detailed of numbers of SNPs and IVW results for each taxa

| Gut microbiota | n SNP | IVW | | |
| --- | --- | --- | --- | --- |
|  |  | b value | SE | P value |
| class Actinobacteria | 7 | -0.0005657 | 0.0009349 | 0.5451 |
| class Alphaproteobacteria | 3 | -0.001522 | 0.00124 | 0.2195 |
| class Bacilli | 10 | -0.001221 | 0.00101 | 0.2269 |
| class Bacteroidia | 5 | 0.001162 | 0.001376 | 0.3984 |
| class Betaproteobacteria | 8 | 0.0008046 | 0.001064 | 0.4494 |
| class Clostridia | 8 | 0.00103 | 0.0008874 | 0.2456 |
| class Coriobacteriia | 5 | 0.0004241 | 0.001402 | 0.7623 |
| class Deltaproteobacteria | 7 | -0.0002148 | 0.00129 | 0.8677 |
| class Erysipelotrichia | 8 | -0.0001189 | 0.001069 | 0.9115 |
| class Gammaproteobacteria | 3 | 0.001724 | 0.00209 | 0.4095 |
| class Lentisphaeria | 6 | -0.00007156 | 0.0006278 | 0.9093 |
| class Melainabacteria | 4 | 0.0005785 | 0.001063 | 0.5864 |
| class Methanobacteria | 4 | 0.001305 | 0.0007833 | 0.09575 |
| class Mollicutes | 7 | 0.00008625 | 0.001019 | 0.9325 |
| class Negativicutes | 5 | 0.002521 | 0.001072 | 0.01863 |
| class Verrucomicrobiae | 9 | -0.0004459 | 0.0009477 | 0.638 |
| family Acidaminococcaceae | 4 | 0.00006733 | 0.001191 | 0.9549 |
| family Actinomycetaceae | 2 | -0.001858 | 0.001188 | 0.1179 |
| family Alcaligenaceae | 7 | -0.0007921 | 0.001176 | 0.5004 |
| family Bacteroidaceae | 5 | 0.001663 | 0.001649 | 0.3132 |
| family Bacteroidales S24 7group | 5 | 0.0008203 | 0.0008607 | 0.3405 |
| family Bifidobacteriaceae | 9 | 0.0006456 | 0.001047 | 0.5376 |
| family Clostridiaceae1 | 2 | 0.0005998 | 0.001488 | 0.687 |
| family Clostridiales vadin BB60 group | 10 | -0.000347 | 0.0006964 | 0.6183 |
| family Coriobacteriaceae | 5 | 0.0004241 | 0.001402 | 0.7623 |
| family Defluviitaleaceae | 8 | 0.0006265 | 0.0006907 | 0.3644 |
| family Desulfovibrionaceae | 8 | 0.001181 | 0.0008833 | 0.1812 |
| family Enterobacteriaceae | 3 | 0.0003634 | 0.001505 | 0.8092 |
| family Erysipelotrichaceae | 8 | -0.0001189 | 0.001069 | 0.9115 |
| family Family XI | 7 | 0.0002098 | 0.0004807 | 0.6624 |
| family Family XIII | 6 | -0.0007507 | 0.001681 | 0.6551 |
| family Lachnospiraceae | 8 | -0.001098 | 0.001054 | 0.2976 |
| family Lactobacillaceae | 4 | 0.0001962 | 0.001002 | 0.8448 |
| family Methanobacteriaceae | 4 | 0.001305 | 0.0007833 | 0.09575 |
| family Oxalobacteraceae | 7 | -0.0009366 | 0.0006985 | 0.1799 |
| family Pasteurellaceae | 7 | -0.001397 | 0.0009514 | 0.142 |
| family Peptococcaceae | 8 | -0.0003358 | 0.0007122 | 0.6372 |
| family Peptostreptococcaceae | 9 | 0.0005819 | 0.0008053 | 0.4699 |
| family Porphyromonadaceae | 3 | -0.002529 | 0.001787 | 0.1571 |
| family Prevotellaceae | 9 | -0.0005149 | 0.001297 | 0.6915 |
| family Rhodospirillaceae | 7 | -0.0008359 | 0.0007948 | 0.293 |
| family Rikenellaceae | 10 | -0.0006439 | 0.0008646 | 0.4564 |
| family Ruminococcaceae | 5 | 0.001046 | 0.001014 | 0.3021 |
| family Streptococcaceae | 8 | -0.000611 | 0.001156 | 0.5972 |
| family unknown | 3 | -0.00169 | 0.001106 | 0.1263 |
| family unknown | 9 | -0.0006095 | 0.0005257 | 0.2462 |
| family unknown | 4 | 0.0005797 | 0.001062 | 0.5852 |
| family Veillonellaceae | 9 | 0.0003395 | 0.0009768 | 0.7281 |
| family Verrucomicrobiaceae | 9 | -0.0004452 | 0.000948 | 0.6386 |
| family Verrucomicrobiaceae | 9 | -0.0004452 | 0.000948 | 0.6386 |
| family Victivallaceae | 9 | 0.0007425 | 0.00044 | 0.09151 |
| family Victivallaceae | 9 | 0.0007425 | 0.00044 | 0.09151 |
| genus Actinomyces | 2 | 0.00197 | 0.002266 | 0.3845 |
| genus Adlercreutzia | 4 | -0.0004877 | 0.001019 | 0.6323 |
| genus Akkermansia | 9 | -0.0004437 | 0.0009478 | 0.6397 |
| genus Alistipes | 5 | 0.00003236 | 0.001923 | 0.9866 |
| genus Allisonella | 4 | -0.0001346 | 0.000603 | 0.8234 |
| genus Alloprevotella | 2 | -0.0003439 | 0.0008782 | 0.6954 |
| genus Anaerofilum | 5 | 0.0005295 | 0.0006375 | 0.4062 |
| genus Anaerostipes | 9 | 0.001414 | 0.0009886 | 0.1526 |
| genus Anaerotruncus | 5 | -0.002396 | 0.001332 | 0.07215 |
| genus Bacteroides | 5 | 0.001663 | 0.001649 | 0.3132 |
| genus Barnesiella | 10 | 0.0002138 | 0.0009816 | 0.8276 |
| genus Bifidobacterium | 5 | -0.0001388 | 0.0009264 | 0.8809 |
| genus Bilophila | 6 | 0.0003444 | 0.001998 | 0.8631 |
| genus Butyricicoccus | 2 | -0.002068 | 0.002545 | 0.4166 |
| genus Butyricimonas | 8 | 0.001536 | 0.0009217 | 0.09571 |
| genus Butyrivibrio | 7 | -0.0001188 | 0.0004948 | 0.8102 |
| genus Candidatus Soleaferrea | 4 | -0.0006332 | 0.0008709 | 0.4672 |
| genus Catenibacterium | 4 | 0.001115 | 0.0008537 | 0.1914 |
| genus Christensenellaceae R 7group | 3 | -0.001205 | 0.001749 | 0.4908 |
| genus Clostridium innocuum group | 2 | 0.002068 | 0.001868 | 0.2682 |
| genus Clostridium sensustricto1 | 6 | -0.001065 | 0.001005 | 0.289 |
| genus Collinsella | 3 | -0.002384 | 0.001535 | 0.1204 |
| genus Coprobacter | 5 | -0.0004967 | 0.001033 | 0.6306 |
| genus Coprococcus1 | 7 | -0.003294 | 0.001028 | 0.001354 |
| genus Coprococcus2 | 3 | 0.0007068 | 0.001373 | 0.6068 |
| genus Coprococcus3 | 5 | -0.002289 | 0.001199 | 0.05623 |
| genus Defluviitaleaceae UCG011 | 8 | 0.0004764 | 0.0006867 | 0.4878 |
| genus Desulfovibrio | 5 | 0.000001113 | 0.0009644 | 0.9991 |
| genus Dialister | 2 | 0.003723 | 0.001718 | 0.03027 |
| genus Dorea | 5 | 0.001731 | 0.001636 | 0.29 |
| genus Eggerthella | 5 | -0.00002019 | 0.0007198 | 0.9776 |
| genus Eisenbergiella | 5 | -0.0004039 | 0.0007747 | 0.6022 |
| genus Enterorhabdus | 3 | 0.002015 | 0.0009595 | 0.03575 |
| genus Erysipelatoclostridium | 8 | 0.00004224 | 0.0008684 | 0.9612 |
| genus Erysipelotrichaceae UCG003 | 1 | 0.00289 | 0.002008 | 0.1502 |
| genus Escherichia Shigella | 4 | 0.0003575 | 0.001196 | 0.7651 |
| genus Eubacterium brachy group | 4 | 0.000647 | 0.0007324 | 0.377 |
| genus Eubacterium coprostanoligenes group | 6 | -0.00122 | 0.001161 | 0.2934 |
| genus Eubacterium eligens group | 3 | -0.0006546 | 0.001497 | 0.6619 |
| genus Eubacterium fissicatena group | 5 | -0.0003387 | 0.0006461 | 0.6002 |
| genus Eubacterium hallii group | 8 | -0.001323 | 0.0008463 | 0.1181 |
| genus Eubacterium nodatum group | 3 | 0.0004455 | 0.0007067 | 0.5284 |
| genus Eubacterium oxidoreducens group | 4 | -0.001101 | 0.001057 | 0.2977 |
| genus Eubacterium rectale group | 4 | -0.0008188 | 0.002505 | 0.7438 |
| genus Eubacterium ruminantium group | 10 | 0.0006814 | 0.0006093 | 0.2634 |
| genus Eubacterium ventriosum group | 7 | 0.0006008 | 0.001702 | 0.7242 |
| genus Eubacterium xylanophilum group | 7 | 0.003385 | 0.000999 | 0.000702 |
| genus Faecalibacterium | 3 | -0.0008387 | 0.001177 | 0.4763 |
| genus Family XIII AD3011 group | 7 | 0.00115 | 0.0009812 | 0.2414 |
| genus Family XIII UCG001 | 5 | 0.0004943 | 0.001057 | 0.6402 |
| genus Flavonifractor | 4 | -0.00002471 | 0.001364 | 0.9855 |
| genus Fusicatenibacte | 9 | -0.000288 | 0.0009807 | 0.769 |
| genus Gordonibacter | 3 | -0.0008624 | 0.0007715 | 0.2636 |
| genus Haemophilus | 9 | -0.0008208 | 0.0008296 | 0.3225 |
| genus Holdemania | 6 | 0.0002667 | 0.000831 | 0.7482 |
| genus Howardella | 7 | 0.0001272 | 0.0005222 | 0.8076 |
| genus Hungatella | 2 | 0.001225 | 0.001009 | 0.2248 |
| genus Intestinibacter | 7 | -0.001069 | 0.0009059 | 0.2382 |
| genus Intestinimonas | 11 | 0.0004506 | 0.0007667 | 0.5567 |
| genus Lachnoclostridium | 6 | 0.0004762 | 0.001233 | 0.6994 |
| genus Lachnospira | 1 | 0.005696 | 0.002931 | 0.05197 |
| genus Lachnospiraceae FCS020 group | 7 | -0.0002761 | 0.0008524 | 0.746 |
| genus Lachnospiraceae NC2004 group | 3 | -0.0000109 | 0.001022 | 0.9915 |
| genus Lachnospiraceae ND3007 group | 1 | -0.002689 | 0.002712 | 0.3214 |
| genus Lachnospiraceae NK4A136 group | 7 | -0.0001425 | 0.0009763 | 0.8839 |
| genus Lachnospiraceae UCG001 | 7 | -0.0009322 | 0.00082 | 0.2556 |
| genus Lachnospiraceae UCG004 | 7 | 0.00009491 | 0.001031 | 0.9267 |
| genus Lachnospiraceae UCG008 | 8 | -0.0001795 | 0.0006498 | 0.7823 |
| genus Lachnospiraceae UCG010 | 5 | 0.0003587 | 0.001581 | 0.8205 |
| genus Lactobacillus | 7 | 0.0005123 | 0.0007129 | 0.4724 |
| genus Lactococcus | 6 | 0.0002019 | 0.0005912 | 0.7328 |
| genus Marvinbryantia | 6 | -0.0006112 | 0.0009868 | 0.5357 |
| genus Methanobrevibacter | 3 | 0.001948 | 0.0007719 | 0.01162 |
| genus Odoribacter | 3 | -0.002322 | 0.001705 | 0.1734 |
| genus Olsenella | 7 | 0.0003326 | 0.0005285 | 0.5292 |
| genus Oscillibacter | 12 | 0.00008102 | 0.000593 | 0.8913 |
| genus Oscillospira | 3 | 0.0001033 | 0.002191 | 0.9624 |
| genus Oxalobacter | 8 | -0.0003245 | 0.0006323 | 0.6078 |
| genus Parabacteroides | 3 | 0.0009558 | 0.001718 | 0.5781 |
| genus Paraprevotella | 9 | 0.00009813 | 0.0006366 | 0.8775 |
| genus Parasutterella | 11 | -0.0001539 | 0.0007124 | 0.8289 |
| genus Peptococcus | 8 | -0.0005014 | 0.000585 | 0.3914 |
| genus Phascolarctobacterium | 6 | 0.0003146 | 0.001063 | 0.7673 |
| genus Prevotella7 | 5 | -0.00002759 | 0.0005971 | 0.9632 |
| genus Prevotella9 | 6 | 0.0008782 | 0.0008074 | 0.2767 |
| genus Rikenellaceae RC9 gut group | 6 | 0.0002857 | 0.0008752 | 0.7441 |
| genus Romboutsia | 3 | 0.001109 | 0.001439 | 0.4409 |
| genus Roseburia | 6 | -0.001372 | 0.001395 | 0.3255 |
| genus Ruminiclostridium5 | 6 | 0.001421 | 0.001241 | 0.252 |
| genus Ruminiclostridium6 | 8 | 0.001409 | 0.0008171 | 0.08458 |
| genus Ruminiclostridium9 | 5 | 0.0008004 | 0.001554 | 0.6065 |
| genus Ruminococcaceae NK4A214 group | 9 | 0.0009945 | 0.0009875 | 0.3139 |
| genus Ruminococcaceae UCG002 | 10 | 0.0002195 | 0.0008338 | 0.7923 |
| genus Ruminococcaceae UCG003 | 10 | 0.0005015 | 0.0008387 | 0.5499 |
| genus Ruminococcaceae UCG004 | 4 | -0.0005101 | 0.001162 | 0.6608 |
| genus Ruminococcaceae UCG005 | 9 | -0.0005187 | 0.0008572 | 0.5451 |
| genus Ruminococcaceae UCG009 | 6 | 0.0002649 | 0.0008166 | 0.7456 |
| genus Ruminococcaceae UCG010 | 4 | 0.0002808 | 0.001285 | 0.827 |
| genus Ruminococcaceae UCG011 | 6 | -0.000908 | 0.0005946 | 0.1268 |
| genus Ruminococcaceae UCG013 | 7 | -0.0007372 | 0.0009591 | 0.4421 |
| genus Ruminococcaceae UCG014 | 6 | 0.0006733 | 0.0009685 | 0.4869 |
| genus Ruminococcus gauvreauii group | 7 | 0.0006315 | 0.0009593 | 0.5103 |
| genus Ruminococcus gnavus group | 7 | -0.0001598 | 0.0007937 | 0.8404 |
| genus Ruminococcus torques group | 5 | -0.001105 | 0.002026 | 0.5853 |
| genus Ruminococcus1 | 5 | -0.0005841 | 0.001743 | 0.7375 |
| genus Ruminococcus2 | 7 | 0.0009047 | 0.0009384 | 0.335 |
| genus Sellimonas | 5 | 0.0002127 | 0.0005623 | 0.7052 |
| genus Senegalimassilia | 2 | -0.003588 | 0.00158 | 0.02319 |
| genus Slackia | 4 | 0.001649 | 0.0009621 | 0.0866 |
| genus Streptococcus | 11 | -0.0002669 | 0.001079 | 0.8046 |
| genus Subdoligranulum | 7 | 0.001396 | 0.001077 | 0.1951 |
| genus Sutterella | 4 | -0.00127 | 0.001244 | 0.3074 |
| genus Terrisporobacter | 2 | -0.0003401 | 0.001144 | 0.7662 |
| genus Turicibacter | 6 | 0.000911 | 0.000911 | 0.4685 |
| genus Tyzzerella3 | 9 | -0.0009425 | 0.0006057 | 0.1197 |
| genus unknown | 8 | 0.0008418 | 0.0007253 | 0.2458 |
| genus unknown | 5 | -0.0003175 | 0.0009345 | 0.734 |
| genus unknown | 6 | 0.0001512 | 0.0007634 | 0.843 |
| genus unknown | 5 | 0.0008203 | 0.0008607 | 0.3405 |
| genus unknown | 4 | 0.0005352 | 0.00138 | 0.6981 |
| genus unknown | 10 | -0.000347 | 0.0006964 | 0.6183 |
| genus unknown | 9 | -0.0008378 | 0.0008331 | 0.3146 |
| genus unknown | 3 | -0.00169 | 0.001106 | 0.1263 |
| genus unknown | 4 | 0.0005797 | 0.001062 | 0.5852 |
| genus Veillonella | 4 | -0.002546 | 0.001393 | 0.06762 |
| genus Victivallis | 5 | -0.0003842 | 0.00106 | 0.7169 |
| genus unknown | 7 | -0.002213 | 0.0008723 | 0.01117 |
| genus unknown | 5 | 0.0009036 | 0.0007569 | 0.2325 |
| order Actinomycetales | 2 | -0.001861 | 0.001191 | 0.118 |
| order Bacillales | 4 | -0.0004053 | 0.0006507 | 0.5334 |
| order Bacteroidales | 5 | 0.001162 | 0.001376 | 0.3984 |
| order Bifidobacteriales | 5 | 0.0006456 | 0.001047 | 0.5376 |
| order Clostridiales | 8 | 0.001032 | 0.000888 | 0.2453 |
| order Coriobacteriales | 5 | 0.0004241 | 0.001402 | 0.7623 |
| order Desulfovibrionales | 8 | 0.001184 | 0.0008856 | 0.1813 |
| order Enterobacteriales | 3 | 0.0003634 | 0.001505 | 0.8092 |
| order Erysipelotrichales | 8 | -0.0001189 | 0.001069 | 0.9115 |
| order Gastranaerophilales | 4 | 0.0005797 | 0.001062 | 0.5852 |
| order Lactobacillales | 9 | -0.001313 | 0.001095 | 0.2303 |
| order Methanobacteriales i | 4 | 0.001305 | 0.0007833 | 0.09575 |
| order Mollicutes RF9 | 3 | -0.00169 | 0.001106 | 0.1263 |
| order NB1n | 9 | -0.0006095 | 0.0005257 | 0.2462 |
| order Pasteurellales | 7 | -0.001397 | 0.0009514 | 0.142 |
| order Rhodospirillales | 9 | -0.0005353 | 0.0007085 | 0.4499 |
| order Selenomonadales | 7 | 0.002521 | 0.001072 | 0.01863 |
| order Verrucomicrobiales | 10 | -0.0004459 | 0.0009477 | 0.638 |
| order Victivallales | 6 | -0.00007156 | 0.0006278 | 0.9093 |
| phylum Actinobacteria | 9 | -0.0002484 | 0.0009927 | 0.8024 |
| phylum Bacteroidetes | 5 | 0.002016 | 0.001374 | 0.1424 |
| phylum Cyanobacteria | 4 | -0.001486 | 0.0008665 | 0.08624 |
| phylum Euryarchaeota | 6 | 0.0002048 | 0.0007006 | 0.77 |
| phylum Firmicutes | 6 | 0.0008451 | 0.001267 | 0.5046 |
| phylum Lentisphaerae | 5 | -0.00005782 | 0.0007623 | 0.9395 |
| phylum Proteobacteria | 4 | -0.0006022 | 0.001402 | 0.6675 |
| phylum Tenericutes | 7 | 0.00008625 | 0.001019 | 0.9325 |
| phylum Verrucomicrobia | 8 | -0.001033 | 0.0011 | 0.3477 |
