# Supplementary material for: The causality between gut microbiota and endometriosis: a bidirectional Mendelian randomization study
Source: Front Med (Lausanne). 2024 Nov 22;11:1434582. doi: 10.3389/fmed.2024.1434582 (PMC11621931; doi:10.3389/fmed.2024.1434582)
Supplement: Supplementary file 2 [file Table_2.DOC]

Table S2. primary causality of gut microbiota on risk of EMs

| exposure | n SNP | IVW | | | MR Egger | | | Weighted median | | | horizontal pleiotropy | | | Heterogeneity | | Causal direction | | |
| --- | --- | --- | --- | --- | --- | --- | --- | --- | --- | --- | --- | --- | --- | --- | --- | --- | --- | --- |
|  |  | b | SE | P-val | b | SE | P-val | b | SE | P-val | ERI | SE | P-val | Q | P-val | WEIE | WEIO | P-val |
| class Negativicutes | 7 | 0.002521 | 0.001072 | 0.01863 | 0.005405 | 0.003049 | 0.1364 | 0.002102 | 0.001445 | 0.1458 | -0.00022 | 0.00022 | 0.359 | 6.198 | 0.4014 | 0.013 | 3.3e-05 | 1e-36 |
| genus Dialister | 2 | 0.003723 | 0.001718 | 0.03027 | - | - | - | - | - | - | - | - | - | 0.7413 | 0.3893 | 0.0032 | 0.0032 | 5.68e-10 |
| genus Enterorhabdus | 3 | 0.002015 | 0.0009595 | 0.03575 | 0.0005135 | 0.002386 | 0.8651 | 0.001771 | 0.001182 | 0.134 | 0.00026 | 0.00038 | 0.617 | 0.7286 | 0.6947 | 0.0088 | 3.2e-05 | 4.01e-25 |
| genus Eubacterium xylanophilum | 7 | 0.003385 | 0.000999 | 0.0007027 | -0.001805 | -0.001805 | 0.5773 | 0.003223 | 0.001374 | 0.01897 | 0.00044 | 0.00025 | 0.131 | 6.42 | 0.3778 | 0.012 | 6.8e-05 | 3.69e-34 |
| genus Methanobrevibacter | 3 | 0.001948 | 0.0007719 | 0.0007719 | 0.002244 | 0.003202 | 0.6108 | 0.001635 | 0.001025 | 0.1107 | -5e-05 | 0.00051 | 0.938 | 1.767 | 0.4134 | 0.0047 | 2.3e-05 | 7.72e-14 |
| order Selenomonadales | 7 | 0.002521 | 0.001072 | 0.01863 | 0.005405 | 0.003049 | 0.1364 | 0.1364 | 0.001445 | 0.1458 | -0.00022 | 0.00022 | 0.359 | 6.198 | 0.4014 | 0.013 | 3.3e-05 | 1e-36 |
| genus Coprococcus 1 | 7 | -0.003294 | 0.001028 | 0.001354 | -0.001902 | 0.003123 | 0.5692 | -0.002869 | 0.001362 | 0.03517 | -0.00011 | 0.00022 | 0.657 | 4.788 | 0.5713 | 0.013 | 4.7e-05 | 4.34e-36 |
| genus Senegalimassilia | 2 | -0.003588 | 0.00158 | 0.02319 | - | - | - | - | - | - | - | - | - | 1.34 | 0.2471 | 0.0062 | 2.8e-05 | 8.48e-18 |
|  |  |  |  |  |  |  |  |  |  |  |  |  |  |  |  |  |  |  |

WEIE=Variance explained in exposure, WEIO=Variance explained in outcome, SE=Standard error, ERI=Egger regression intercept
