# Supplementary material for: The causality between gut microbiota and endometriosis: a bidirectional Mendelian randomization study
Source: Front Med (Lausanne). 2024 Nov 22;11:1434582. doi: 10.3389/fmed.2024.1434582 (PMC11621931; doi:10.3389/fmed.2024.1434582)
Supplement: Supplementary file 3 [file Table_3.DOC]

Table S3. [Verified](javascript:;) causality of gut microbiota on risk of EMs

| exposure | n SNP | IVW | | | MR Egger | | | Weighted median | | | horizontal pleiotropy | | | Heterogeneity | |
| --- | --- | --- | --- | --- | --- | --- | --- | --- | --- | --- | --- | --- | --- | --- | --- |
|  |  | b | SE | P-val | b | SE | P-val | b | SE | P-val | ERI | SE | P-val | Q | P-val |
| phylum Cyanobacteria | 4 | 0.2114 | 0.001072 | 0.03997 | 0.005405 | 0.3862 | 0.4574 | 0.2096 | 0.1204 | 0.08151 | -0.026 | 0.062 | 0.359 | 0.3783 | 0.4014 |
| genus Ruminococcaceae UCG002 | 10 | 0.2942 | 0.114 | 0.009893 | 0.3426 | 0.3426 | 0.3082 | 0.2944 | 0.1335 | 0.02741 | -0.0041 | 0.025 | 0.025 | 13.77 | 0.1308 |
| genus Coprococcus3 | 5 | 0.3251 | 0.1539 | 0.03467 | 0.133 | 0.7156 | 0.8644 | 0.3271 | 0.2013 | 0.1042 | 0.012 | 0.044 | 0.801 | 2.29 | 0.6825 |
| genus Bifidobacterium | 6 | -0.2059 | 0.09133 | 0.02419 | -0.2807 | 0.2221 | 0.2749 | -0.2196 | 0.1219 | 0.07167 | 0.0084 | 0.023 | 0.73 | 3.512 | 0.6216 |
| genus Flavonifractor | 4 | -0.2742 | 0.1345 | 0.04152 | -0.4629 | 0.7113 | 0.7113 | -0.2722 | 0.1625 | 0.09396 | 0.015 | 0.054 | 0.812 | 1.619 | 0.655 |
| genus Rikenellaceae RC9 | 6 | -0.1489 | 0.06494 | 0.02187 | 0.1555 | 0.4107 | 0.7241 | -0.1218 | 0.08008 | 0.1282 | -0.043 | 0.058 | 0.494 | 5.667 | 0.34 |

SE=Standard error, ERI=Egger regression intercept
