# Supplementary material for: The causality between gut microbiota and endometriosis: a bidirectional Mendelian randomization study
Source: Front Med (Lausanne). 2024 Nov 22;11:1434582. doi: 10.3389/fmed.2024.1434582 (PMC11621931; doi:10.3389/fmed.2024.1434582)
Supplement: Supplementary file 4 [file Table_4.DOC]

Table S4. Primary causality of EMs on gut microbiota

| outcome | n SNP | IVW | | | MR Egger | | | Weighted median | | | horizontal pleiotropy | | | Heterogeneity | | Causal direction | | |
| --- | --- | --- | --- | --- | --- | --- | --- | --- | --- | --- | --- | --- | --- | --- | --- | --- | --- | --- |
|  |  | b | SE | P-val | b | SE | P-val | b | SE | P-val | ERI | SE | P-val | Q | P-val | WEIE | WEIO | P-val |
| genus Ruminococcaceae UCG009 | 7 | 28.39 | 8.486 | 0.0008221 | 48.12 | 39.44 | 0.2769 | 29.9 | 11.47 | 0.009118 | -0.022 | 0.042 | 0.63 | 4.308 | 0.635 | 0.013 | 0.0011 | 0.285 |
| genus  Eubacterium  fissicatena | 6 | -37.79 | 12.67 | 0.002848 | 33.16 | 70.21 | 0.6614 | -41.13 | 16.45 | 0.01241 | -0.074 | 0.073 | 0.362 | 4.706 | 0.4528 | 0.00054 | 0.001 | 0.287 |
| genus Prevotella7 | 6 | -33.52 | 15.03 | 0.02578 | -116.4 | 83.09 | 0.2337 | -26.84 | 17.7 | 0.1295 | 0.087 | 0.086 | 0.368 | 6.607 | 0.2515 | 0.00054 | 0.0011 | 0.259 |
| genus Butyricicoccus | 7 | -11.23 | 5.632 | 0.04619 | 31.2 | 26.17 | 0.2866 | -10.2 | 7.588 | 0.179 | -0.046 | 0.028 | 0.158 | 4.105 | 0.6624 | 0.00061 | 0.00083 | 0.63 |
| family Lactobacillaceae | 7 | -18.8 | 8.866 | 0.03396 | 6.417 | 41.42 | 0.883 | -25.52 | 11.01 | 0.02051 | -0.028 | 0.044 | 0.56 | 2.696 | 0.8459 | 0.00061 | 0.00056 | 0.911 |

WEIE=Variance explained in exposure, WEIO=Variance explained in outcome, SE=Standard error, ERI=Egger regression intercept
