# Supplementary material for: The causality between gut microbiota and endometriosis: a bidirectional Mendelian randomization study
Source: Front Med (Lausanne). 2024 Nov 22;11:1434582. doi: 10.3389/fmed.2024.1434582 (PMC11621931; doi:10.3389/fmed.2024.1434582)
Supplement: Supplementary file 5 [file Table_5.DOC]

Table S5. Verified causality of EMs on gut microbiota

| outcome | n SNP | IVW | | | MR Egger | | | Weighted median | | | horizontal pleiotropy | | | Heterogeneity | |
| --- | --- | --- | --- | --- | --- | --- | --- | --- | --- | --- | --- | --- | --- | --- | --- |
|  |  | b | SE | P-val | b | SE | P-val | b | SE | P-val | ERI | SE | P-val | Q | P-val |
| genus Howardella | 19 | 0.1271 | 0.04989 | 0.01087 | -0.07908 | 0.1896 | 0.6818 | 0.04372 | 0.06991 | 0.06991 | 0.026 | 0.023 | 0.275 | 16.75 | 0.5403 |
| genus Dialister | 19 | -0.07478 | 0.03145 | 0.01742 | -0.1765 | 0.1197 | 0.1197 | -0.07082 | 0.04478 | 0.1138 | 0.013 | 0.015 | 0.391 | 0.7413 | 0.4917 |

SE=Standard error, ERI=Egger regression intercept
